# Conjugated linoleic acid induces apoptosis through estrogen receptor alpha in human breast tissue

**DOI:** 10.1186/1471-2407-8-208

**Published:** 2008-07-24

**Authors:** Li-Shu Wang, Yi-Wen Huang, Suling Liu, Pearlly Yan, Young C Lin

**Affiliations:** 1 Department of Veterinary Biosciences, College of Veterinary Medicine, The Ohio State University, Columbus, OH 43210, USA; 2 OSU Comprehensive Cancer Center, The Ohio State University, Columbus, OH 43210, USA; 3 University of Michigan Comprehensive Cancer Research Center, Ann Arbor, MI 48109, USA; 4 Laboratory of Reproductive and Molecular Endocrinology, College of Veterinary Medicine, The Ohio State University, Columbus, OH, USA

## Abstract

**Background:**

Conjugated linoleic acid (CLA), a naturally occurring fatty acid found in ruminant products such as milk and beef, has been shown to possess anti-cancer activities in *in vivo *animal models and *in vitro *cell culture systems. In human breast cancer, the overall duration of estrogen exposure is the most important risk factor for developing estrogen-responsive breast cancer. Accordingly, it has been suggested that estrogen exposure reduces apoptosis through the up-regulation of the anti-apoptosis protein, Bcl-2. Bcl-2, an anti-apoptotic protein, regulates apoptosis and plays a crucial role in the development and growth regulation of normal and cancerous cells. Our research interest is to examine the effects of CLA on the induction of apoptosis in human breast tissues.

**Methods:**

The localization of Bcl-2 in both normal and cancerous human breast tissues was determined by immunohistochemical staining and the Bcl-2 protein expression was tested by western blot analysis. Co-culture of epithelial cells and stromal cells was carried out in the presence or absence of CLA to evaluate apoptosis in the context of a cell-cell interaction.

**Results:**

The results showed that both normal and cancerous breast tissues were positive for Bcl-2 staining, which was higher overall in mammary ducts but very low in the surrounding stromal compartment. Interestingly, by quantifying the western blot data, basal Bcl-2 protein levels were higher in normal breast epithelial cells than in cancerous epithelial cells. Furthermore, treatment with 17β-estradiol (E_2_) stimulated growth and up-regulated Bcl-2 expression in estrogen responsive breast epithelial cells; however, these carcinogenic effects were diminished by either CLA or 4-Hydroxytamoxifen (Tam) and were suppressed further by the combination of CLA and Tam. In both one cell type cultured and co-culture systems, CLA induced cell apoptosis in ERα transfected MDA-MB-231 cells but not in the wild type MDA-MB-231 cells.

**Conclusion:**

These data, therefore, demonstrate that ERα plays important roles in CLA induced apoptosis in human breast tissues.

## Background

CLA is produced by rumen fermentation of linoleic acid and is deposited in the subcutaneous fat and intramuscular fat layer in beef cattle; it also is present in dairy milk fat [1]. There are several CLA isomers in ruminant-produced foods, among them *c*9, *t*11-CLA and *t*10, *c*12-CLA are more potent against tumor cell growth *in vitro *[2]. Furthermore, rats fed CLA-enriched butter fat had reduced mammary cancer risk [2]. Studies also showed that beef CLA isomers even when associated with other beef fatty acids reduce human cancer cell growth [3] and beef tallow increases the potency of CLA against mouse mammary tumor metastasis [4]. CLA studies in our laboratory [5-7] demonstrated the anti-tumor effect of CLA (I) on angiogenesis by suppression of the predominant vascular endothelial growth factor (VEGF) isoforms, VEGF 121 and 165, and mRNA expression in a human breast cancer cell line; (II) via up-regulation of the estrogen-regulated cancer suppressor gene, protein tyrosine phosphatase γ (PTPγ) in human breast cells; and (III) by modulation of prostaglandin E_2 _(PGE_2_) signaling in canine mammary cells.

Potential modulation of apoptosis by anti-cancer agents is of interest for several reasons. Apoptosis, programmed cell death, is thought to play a key role in the development and growth regulation of normal and cancerous cells. Consequently, dysregulation of apoptosis can result in carcinogenesis [8]. The regulation of apoptosis involves a large set of proteins including Bcl-2, an anti-apoptotic protein [8]. In human breast cancers, about 75% of breast cancers are estrogen receptor α (ERα) positive. Estrogens cause initiation, promotion, and progression of these tumors. E_2 _is the most abundant circulating endogenous estrogen [9,10]. In the rat model, higher Bcl-2 expression is detected in mammary epithelial cells when animals are treated with E_2 _[11]. The anti-estrogen tamoxifen is the most frequently prescribed drugs for estrogen responsive breast cancer patients and also is recommended for women at high risk of developing breast cancer [12]. Tamoxifen therapy generally is well tolerated and leads to prolonged cancer-free survival and decreased mortality even in patients with ER-positive metastatic tumors [13,14]. However, the development of tamoxifen resistance and the incidence of developing endometrial cancer after five-year tamoxifen therapy is a critical issue [12,15]. Updated data from the Arimidex, Tamoxifen, alone or in Combination (ATAC) trial showed that the aromatase inhibitor appeared to be superior to tamoxifen in postmenopausal women with ER-positive breast cancer [16]. Nevertheless, aromatase inhibitors cause different side effects compared with those of tamoxifen, and the greater level of the toxicity versus the efficacy of aromatase inhibitors needs to be investigated further [17]. Thus, the American Society of Clinical Oncology (ASCO) Health Services Research Committee recommends that patients intolerant to aromatase inhibitors should receive tamoxifen. The concern in using endocrine therapy for chemopreventive and/or chemotherapeutic purposes guided us to investigate naturally existing compounds in food that possess anticancer activity, such as CLA. Daily consumption of CLA from ruminant-based foods for cancer prevention will be less likely to raise concerns about toxicity. However, more recent data raised the concern about safety issue of CLA. Dietary CLA, mainly *t*10, *c12*-CLA, induced insulin resistance and increased plasma insulin levels in both normal and obesity mice, and more important, similar results have been reported in recent clinical trials [2]. It has also been demonstrated that high level of *t*10, *c12*-CLA induced liver steatosis in mice [2]. Thus use this isomer in the clinical need to be very cautious.

It has been theorized that the paracrine signals produced by surrounding stromal cells plays a crucial role in modulating malignant epithelial cells progression [18,19]. Studies suggest that CLA is incorporated, stored in stromal cells, and CLA affect cancer progression [2]. CLA and human breast cancer study revealed that dietary CLA intake was associated with the regulation of estrogen receptor expression *in vivo *[20]. CLA has been found to reduce the risk of developing an estrogen receptor-negative tumor in premenopausal women, which may lead to a better therapeutic outcome for breast cancer patients as their cancer will be likely responsive to anti-estrogen therapy [20]. Based on this epidemiological finding, we hypothesize that CLA will exert anti-cancer activities in ERα-positive breast tumors. In this study, we examined the role of ERα in CLA-induced apoptosis in human breast epithelial cells and the possible involvement of apoptotic marker, Bcl-2, on CLA-mediated apoptosis.

## Methods

### Reagents

*t*10, *c*12-CLA (> 98% pure), the CLA isomer used in this study, was purchased from Matreya, Inc. (Greenland, NH) and a CLA stock solution was prepared as described previously [21]. For the steroid hormone study, we used Dextran-Coated Charcoal (DCC, Dextran T-70; Pharmacia; activated charcoal; Sigma, St. Louis, MO) to remove steroid hormones in the fetal bovine serum (FBS, GibcoBRL, Bethesda, MD). We then added the CLA to medium with the DCC-treated FBS. 4-Hydroxytamoxifen (Tam), docetaxel and 17β-estradiol (E_2_) were purchased from Sigma (St. Louis, MO).

### Immortalized cell line

MCF-7 and MDA-MB-231 cells were purchased from American Type Culture Collection (ATCC, Manassas, VA). MDA-MB-231-ERα cells (MDA-MB-231 cells stably transfected with ERα) were a gift from Dr. Robert Brueggemeier at the College of Pharmacy, The Ohio State University. All cell lines were cultured in phenol-red free high-calcium Dulbecco's Modified Eagle's Medium and Ham's F12 Medium (DMEM/F12, 1.05 mM CaCl_2_) supplemented with 5% fetal bovine serum (FBS) in 75 cm^2 ^flasks and incubated in a humidified incubator (5% CO_2_, 95% air, 37°C). The medium was renewed every two days. Geneticin (500 mg/ml) was used every 2–3 passages for maintenance and selection of MDA-MB-231-ERα cells.

### Isolation of epithelial and stromal cells from human breast tissues

Normal (reduction mammoplastys) and malignant human breast tissues were obtained through the Tissue Procurement Program at The Ohio State University Hospital in Columbus, Ohio. Tissues were placed in DMEM/F12 and stored at 4°C. Isolation of epithelial and stromal cells from human breast tissues and culture conditions were described previously [5,6]. Briefly, tissues were minced and digested in 0.1% collagenase I (GibcoBRL, Bethesda, MD) supplemented with 5% FBS and antibiotic-antimycotic (100 unit/ml penicillin G sodium, 100 mg/ml amphotericin B) (GibcoBRL, Bethesda, MD) in 37°C humidified incubator (5% CO_2_: 95% air) overnight. The digested mixture was centrifuged at 200 × g for 5 min at 25°C. The cell pellet was re-suspended and allowed to settle by gravity for 3 times. Stromal cells in the supernatant was then centrifuged at 200 × g for 5 min at 25°C and the pelleted stromal cells were re-suspended in phenol red-free high-calcium DMEM/F12 (1.05 mM CaCl_2_) supplemented with 5% FBS. Cancer epithelial cells in the initial sedimented part were re-suspended in keratinocyte serum free medium (Keratinocyte-SFM, 0.09 mM CaCl_2_) (GibcoBRL) supplemented with bovine pituitary extract (25 mg) and epidermal growth factor (10 μg). Normal epithelial cells initial sedimented part were re-suspended in low calcium DMEM/F12 (0.04 mM CaCl_2_) supplemented with Chelex-100 (Bio-Rad Laboratories, Richmond, CA) -treated FBS (10%).

### Treatment

Unless otherwise stated, cells were cultured in 5% DCC-containing medium for two days followed by treatment with CLA and/or E_2 _and/or Tam in the same medium for three days. Finally, cells were harvested for performing assays described in the following sections.

### Co-culture system

Co-cultures of epithelial cells, MCF-7, MDA-MB-231 or MDA-MB-231-ERα, with stromal cells, non-cancerous or cancerous breast stromal cells, were performed using flat-bottomed cell culture plates with the nucleopore polycarbonate membrane (0.4 μm pore size) of the cell culture inserts. Culture condition for co-culture system was described previously [5]. At the end of the treatment, epithelial cells on the bottom chamber were subjected to determine proliferation by caspase-3/7 activity assay and Bcl-2 protein expression by western blot analysis as described in the following sections.

### Western blot analysis

At the end of the treatment period, cells were washed with ice-cold PBS and then lysed with extraction reagent (Pierce, Rockford, IL) and protease inhibitor (Pierce, Rockford, IL) on ice. The performance of western blot analysis was described previously [5]. Bcl-2 rabbit polyclonal antibody (sc-492, Santa Cruz, CA,) and β-actin goat polyclonal antibody (sc-1615, Santa Cruz, CA,) were utilized in this experiment.

### Non-radioactive cell proliferation assay

Cell proliferation was quantified using the CellTiter 96™ AQueous assay (Promega, Madison, WI). This assay measures the amount of dehydrogenase enzymes found in metabolically active cells by adding enzyme substrate (MTS, 3-(4,5-dimethylthiazol-2-yl)-5-(3-carboxymethoxyphenyl)-2-(4-sulfophenyl)-2H-tetrazolium, inner salt) and electron coupling reagent (PMS, phenazine methosulfate). Briefly, 1 × 10^4 ^cells in 100 μl medium were seeded and treated in 96-well plate. Then, 20 μl of MTS:PMS (20:1) solution was added to each well. Plates were incubated at 37°C for 1.5 hours and the color density was checked every 30 minutes. Finally, optical density was read at 490 nm (OD490 nm) using an ELISA plate reader.

### Hoechst staining for apoptotic cells

Hoechst 33342, a DNA intercalating dye, fluoresces blue when bound to DNA. For this part of the study, 2 × 10^4 ^cells were seeded on coverslips in triplicate. At the end of treatment, cells on the coverslips were washed with 1X PBS, fixed with 4% paraformaldehyde and stained with 5 mg/ml of Hoechst 33342 (Promega, Madison, WI) for 30 min at room temperature. The cells were washed again with 1X PBS and mounted on glass slides. Under confocal fluorescence microscopy (excitation 350 nm and emission 461 nm), apoptotic cells showed condensed chromatin that was bright blue. Samples were stained and counted in triplicate.

### Immunohistochemical staining

The basal expression of Bcl-2 in 14 normal and 14 cancerous human breast tissue samples were fixed, dehydrated, embedded in paraffin, and sectioned for immunohistochemical staining. Immunostaining was carried out using the Vectastain Universal Quick kit (Vector Laboratories, Burlingame, CA) followed by the DAB Substrate Kit for Peroxidase (Vector Laboratories, Burlingame, CA) according to manufacturer's instructions. After stopping the reaction with water, the specimens were counterstained (Hematoxylin, Vector Laboratories, Burlingame, CA) and mounted (Crystal/Mount, Biomeda, Foster City, CA). Antibody against Bcl-2 is a rabbit polyclonal antibody raised against a peptide mapping at the N-terminus of Bcl-2 of human origin (sc-492 Santa Cruz, CA). To test the specificity of Bcl-2 antibody, the primary antibody was substituted with non-immune serum. Bcl-2 intensity was evaluated by Allred scoring, which is a method that conveys estimated proportion score and intensity score [22]. Scores typically ranged from 0–8 and a score ranking more than 3 (corresponds to ≥ 10% positive cells) was defined as Bcl-2 positive [22].

### Caspase-3/7 activity assay

Caspase-3 and -7 are members of the cysteine aspartic acid-specific protease family and play important roles in apoptosis in mammalian cells [23]. Each well contained 10^4 ^cells in 50 μl of medium were seeded and treated in 96 well-plates. At the end of treatment, caspase-3/7 activity was quantified by Apo-ONE^® ^homogenous caspase-3/7 assay according to the manufacturer's instructions (Promega, Madison, WI). Briefly, 50 μl reagent (fluorometric substrate: buffer in the ratio of 1:100) was added to each well. Plates then were incubated at 37°C for 5 hours and the intensity of the emitted fluorescence was determined with the use of a fluorescence spectrometer (excitation 485 nm and emission 530 nm).

### Statistics

The data for cell proliferation, Hoechst staining, caspase-3/7 activity, and western blot of Bcl-2 protein expression were presented as the mean ± standard deviation (SD) and was analyzed using StatView^® ^ANOVA unpaired *t*-test. p-value less than 0.05 was considered to be statistically significant. One cell group consisted of three replicate wells.

## Results

### CLA potentiates the effects of Tam in MCF-7 (ERα(+)) cells

**Figure 1:**
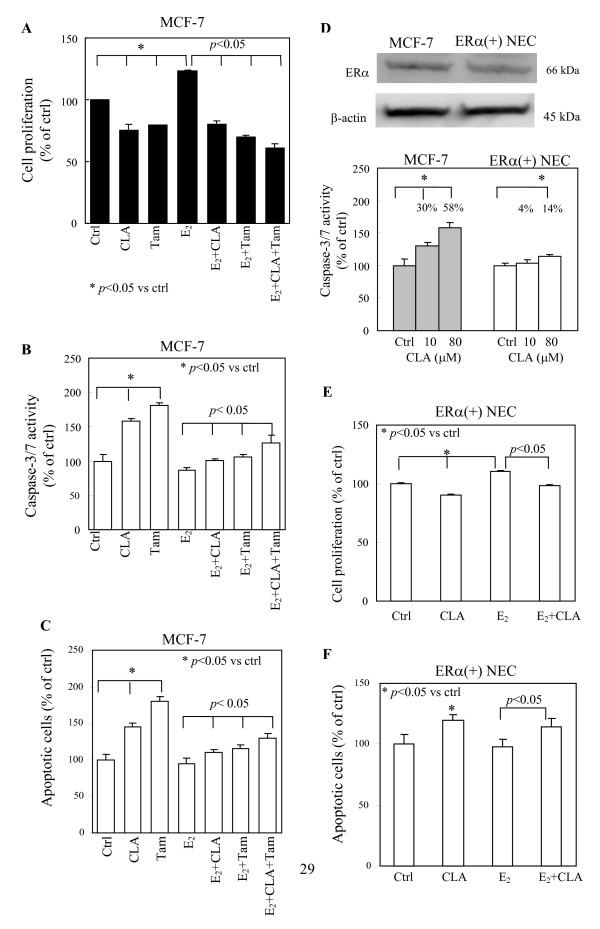
**Effects of CLA on ERα(+) human breast cells.** (A) MTS assay, (B) Apo-ONE^® ^homogeneous caspase-3/7 assay and (C) Hoechst staining for 3 days of treatment with CLA, Tam and E_2 _alone or combined with each other in MCF-7 cells. CLA stands for 40 μM *t*10, *c*12-CLA; Tam stands for 1 μM 4-Hydroxytamoxifen; E_2 _stands for 10 nM 17β-estradiol. CLA exerts combinative effects with tamoxifen in MCF-7 cells. (D) Western blot analysis of basal ERα protein expression in MCF-7 and primary cultured ERα positive normal human breast epithelial cells (ERα(+) NEC). Equal amounts of isolated protein from both cell extracts were subjected to immunoblot with anti-ERα antibodies. β-actin was used as loading control. Histogram: Apo-ONE^® ^homogeneous caspase-3/7 assay for the effects of treatment on apoptosis in MCF-7 and ERα(+) NEC for 3 days of treatment. (E) and (F) showed the effects of CLA and E_2 _on proliferation (MTS assay) and apoptosis (Hoechst staining) in ERα(+) NEC, respectively. 80 μM *t*10, *c*12-CLA; E_2 _stands for 10 nM 17β-estradiol. Bars represent mean ± SD, n = 3. *p < 0.05.

Based on CLA concentration in normal physiologic human serum (10–70 μM) and in humans who take CLA long-term supplementation (50–350 μM), CLA concentrations used in *in vitro *studies has ranged from 12.5–250 μM, the mid-normal to supraphysiologic-pharmacologic levels [24,25]. Our previous study showed that the effective dose range for inhibiting the proliferation of human breast cancerous epithelial and stromal cells was 10–80 μM for three days and *t*10, *c*12-CLA was more potent than *c*9, *t*11-CLA (unpublished data). Therefore, *t*10, *c*12-CLA was used in the current study and a relatively lower but effective dose, 40 μM of *t*10, *c*12-CLA, was chosen to investigate whether CLA potentiates the anti-proliferative and pro-apoptotic effects of Tam. E_2_-stimulated cell growth was suppressed by CLA or Tam, and the combination of CLA with Tam decreased the stimulative effects of E_2 _in the ERα(+) human breast cancer cell line, MCF-7 even further (Fig. 1A). Moreover, we observed that the combination of CLA and Tam caused more apoptosis than treated alone in MCF-7 cells, as measured using apoptotic indicators (Figs. 1B and 1C). These results suggested that CLA may enhance the therapeutic efficiency of Tam in estrogen-responsive human breast cancer patients.

### CLA possesses greater apoptotic effect in ERα(+) breast cancer epithelial cells than in ERα (+) normal breast epithelial cells

Since CLA, a naturally occurring food component, will be consumed by normal individuals as well, we examined the effects of CLA on normal human breast epithelial cells. We first utilized ERα(+) primary cultured normal human breast epithelial cells (ERα(+) NEC) to examine whether CLA also targets this type of cells. The estrogen receptor positivity of the normal breast epithelial cells was determined based on their ERα protein expression. MCF-7 (ERα positive) and MDA-MB-231 (ERα negative) were used as positive and negative control, respectively in the determination of ERα expression in normal breast epithelial cells. CLA was more effective in inducing apoptosis in MCF-7 cells than in ERα(+) NEC (Fig. 1D). 10 μM of CLA did not induce significant changes in apoptosis in ERα(+) NEC. Thus, we used a higher dose of CLA (80 μM) and we observed significant effects on apoptosis in ERα(+) NEC (Fig. 1D). Next, we investigated whether CLA could suppress the mitogenic activity of E_2 _in ERα(+) NEC. CLA treatment suppressed the ability of E_2 _to stimulate proliferation (Fig. 1E) and CLA induced apoptosis in the presence of E_2 _(Fig. 1F). Although the proliferative response of ERα(+) NEC to E_2 _appears to be small, we used Bcl-2 response to confirm that these cells are E_2 _responsive cells (data not shown). These results suggested that CLA may be chemopreventive *in vivo*.

### CLA is more effective in inducing apoptosis in ERα(+) breast cells than in ERα(-) breast cells

**Figure 2:**
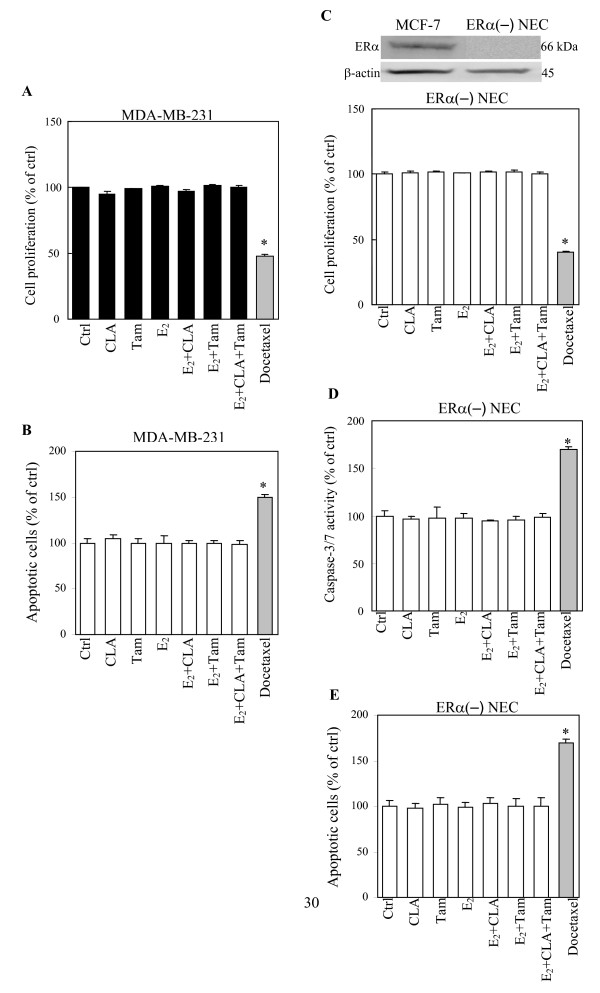
**Effects of CLA, Tam and E_2 _on cell proliferation and apoptosis of ERα negative human breast epithelial cells.** (A) and histogram in (C) are MTS assay for the effects of CLA, Tam and E_2 _for 3 days of treatment on cell proliferation of MDA-MB-231 and ERα negative normal human breast epithelial cells (ERα(-) NEC), respectively. (C) Western blot analysis of basal ERα protein expression in MCF-7 and primary cultured ERα negative normal human breast epithelial cells (ERα(-) NEC). Equal amounts of isolated protein from both cell extracts were subjected to immunoblot with anti-ERα antibodies. β-actin was used as loading control. Hoechst staining of (B) MDA-MB-231 and (E) ERα(-) NEC, and (D) Apo-ONE^® ^homogeneous caspase-3/7 assay of ERα(-) NEC after 3 days of CLA, Tam and E_2 _treatment. CLA stands for 40 μM *t*10, *c*12-CLA. Tam stands for 1 μM 4-Hydroxytamoxifen; E_2 _stands for 10 nM 17β-estradiol. *p < 0.05 versus control. Docetaxel, the commonly used anti-cancer drug, was used as the positive control in the assay of cell proliferation and apoptotic activity.

**Figure 3:**
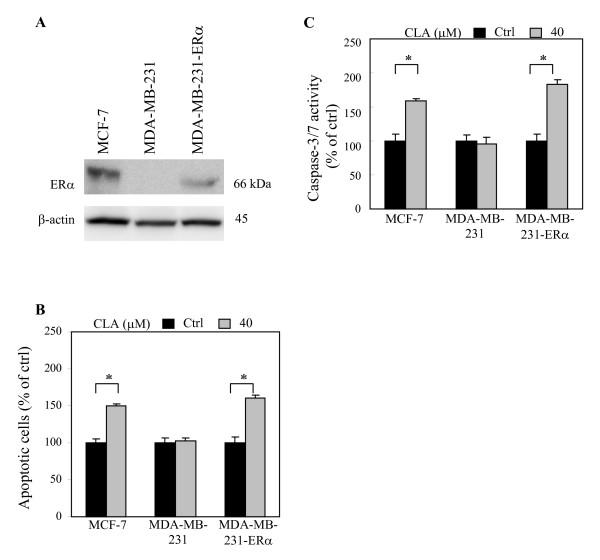
**CLA induces apoptosis in ERα positive human breast cancer cell line.** (A) ERα protein expression was examined by western blot of whole cell lysate isolated from MCF-7, MDA-MB-231 (parental), and MDA-MB-231-ERα (ERα transfected MDA-MB-231). Equal amounts of isolated protein from cell extracts of all three cell lines were subjected to immunoblot with anti-ERα antibodies. β-actin was used as loading control. Effects of CLA on apoptosis of MCF-7, MDA-MB-231, and MDA-MB-231-ERα were determined by Hoechst staining (B) and Apo-ONE^® ^homogeneous caspase-3/7 assay (C). Bars represent mean ± SD, n = 3. *p < 0.05 stands for control versus 40 μM *t*10, *c*12-CLA.

In our *in vitro *system, neither CLA at 40 μM, Tam nor E_2 _caused considerable changes of cell growth or apoptosis in the ERα(-) human breast cancer cell line, MDA-MB-231, (Figs. 2A and 2B) or ERα(-) human primary cultured normal breast epithelial cells (ERα(-) **NEC) **(Figs. 2C, D and 2E). Based on this observation, we suspect that CLA treatment resulted in decreased cell growth and increased cell apoptosis is mainly mediated through ERα. To test this possibility, we examined whether CLA exerted the same effects in MDA-MB-231 stably transfected with ERα (MDA-MB-231-ERα) cells as in MCF-7 cells. Our results showed that CLA induced apoptosis in MCF-7 and MDA-MB-231-ERα cells but did not have significant effects on parental MDA-MB-231 cells (Figs. 3B and 3C).

### CLA inhibited E_2 _stimulation of Bcl-2 expression in ERα(+) breast cells

**Figure 4:**
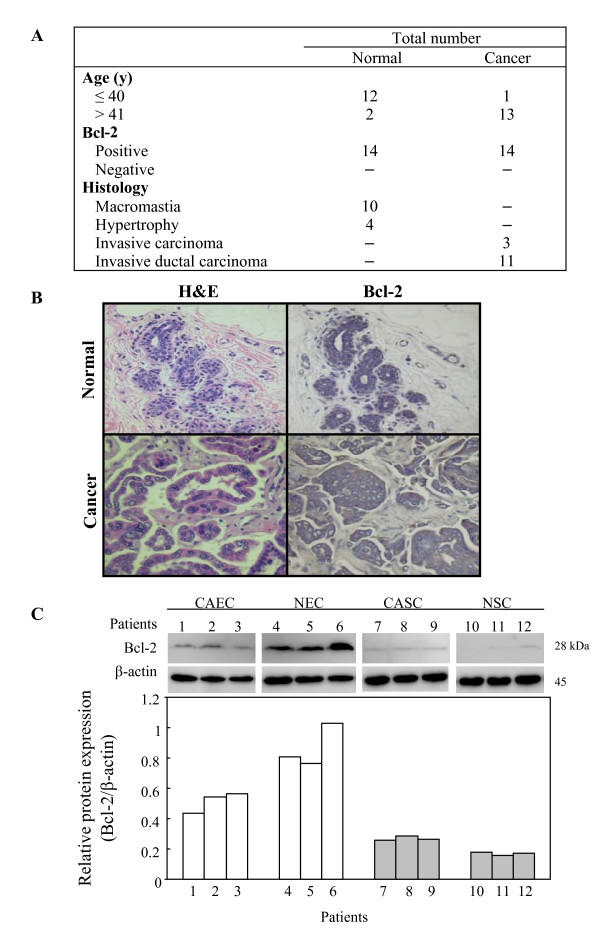
**Bcl-2 expression in human breast.** (A) Patients' information and Bcl-2 immunohistochemistry staining on both normal and cancerous breast tissues from human patients. (B) H&E staining and Bcl-2 immunohistochemistry staining showing dark to brown on one of the normal and one of the cancerous human breast tissues. Blue staining represents nuclei. (C) Western blot analysis of Bcl-2 protein expression in primary cultured human breast cells. CAEC stands for cancer epithelial cells; NEC stands for normal epithelial cells; CASC stands for cancer stromal cells; NSC stands normal stromal cells. Equal amounts of isolated protein from whole cell lysate were subjected to immunoblot with anti-Bcl-2 antibodies. β-actin was used as loading control.

Since Bcl-2 is an anti-apoptotic protein, we would expect to see higher Bcl-2 expression in cancerous human breast tissues. To examine this speculation, immunohistochemical staining for Bcl-2 expression was performed on 14 normal and 14 cancerous human breast tissue samples. In this study, normal patients were younger (≤ 40 years of age) than cancer patients (> 41 years of age) and based on Allred scoring, all tissue samples were positive for Bcl-2 staining (score = 3) (Fig. 4A). Bcl-2 expression was localized to cytoplasm and perinuclear area of cells mainly in mammary ductal epithelium with slight staining of stromal cells surrounding the mammary ducts (Fig. 4B). Under the same magnification, Bcl-2 staining intensity was greater in cancerous tissue compared with normal tissues, which might be due to the larger cytoplasmic volume and larger mammary duct compartment in the malignant samples. To quantify Bcl-2 expression, we isolated epithelial cells and stromal cells from both cancerous and normal breast tissues. Whole cell lysates were electrophoresed and Bcl-2 protein expression was determined by western blot. In agreement with immunohistochemical staining results, Bcl-2 protein was detected in epithelial cells that lined the mammary duct but was very weak in stromal cells (Fig. 4C). Interestingly, Bcl-2 expression was higher in primary cultured normal breast epithelial cells (NEC) than in primary cultured cancerous human breast epithelial cells (CAEC) (Fig. 4C). These results suggest that Bcl-2 may be important in maintaining normal breast function.

**Figure 5:**
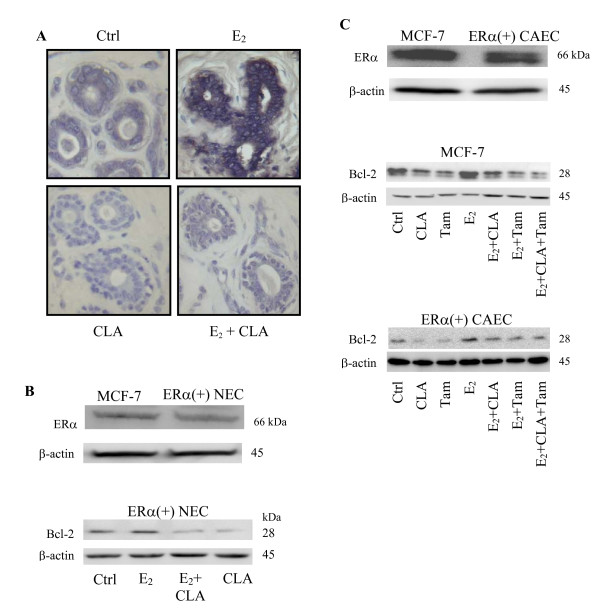
**CLA decreases E_2 _stimulated Bcl-2 protein expression in ERα(+) breast cells.** (A) Immunohistochemistry of Bcl-2 staining (brown to dark) in ERα(+) normal human breast tissue for 3 days of treatment. (B) Western blot analysis of basal ERα protein expression in MCF-7 and ERα(+) NEC, and the effects of CLA and E_2 _on Bcl-2 protein expression in ERα(+) NEC for 3 days of treatment. β-actin was used as loading control. (C) Western blot analysis of basal ERα protein expression in MCF-7 and ERα positive human breast cancer epithelial cells (ERα(+) CAEC). And Bcl-2 protein expression in MCF-7 and ERα(+) CAEC was detected by western blot analysis after 3 days of treatment. CLA exerts combinative effects with Tam in decreasing E_2 _stimulated Bcl-2 protein expression in ERα(+) CAEC. CLA stands for 40 μM *t*10, *c*12-CLA; E_2 _stands for 10 nM 17β-estradiol.

To evaluate whether the effect of CLA on Bcl-2 expression was due to modulation of ERα, ERα(+) normal human breast tissue and cells were treated with CLA, E_2 _or CLA+E_2_. Bcl-2 expression was measured by immunohistochemical staining and western blot. CLA treatment decreased E_2_-stimulated Bcl-2 expression in tissues after three days of treatment (Fig. 5A). This was confirmed by quantification of western blot Bcl-2 protein results (Fig. 5B). Next, we examined whether CLA could potentiate the anti-estrogenic effects of Tam in MCF-7 and ERα(+) CAEC. CLA which is in combination with Tam further suppressed E_2_-induced Bcl-2 protein expression in both MCF-7 (Fig. 5C) and ERα(+) CAEC (Fig. 5C). Interestingly, there are two bands of Bcl-2 were detected by western blot analysis in MCF-7 cells (Fig. 5C) whereas only one band in ERα(+) CAEC (Fig. 5C). Different migration of the same protein may be caused by the post-translational modification, such as phosphorylation. It has been suggested that phosphorylation will stabilize Bcl-2 which may lead to anti-apoptosis of the cell [26,27]. By contrast, de-phosphorylation of Bcl-2 will sensitize cell to apoptosis inducing agent. Thus it is reasonable to speculate that MCF-7 cells, with immortalized characteristic, will be more resistance to apoptosis in comparison with ERα(+) CAEC which is primary cultured from human breast tissue.

### Effects of stromal cells on CLA regulated Bcl-2 expression in human breast epithelial cells

**Figure 6:**
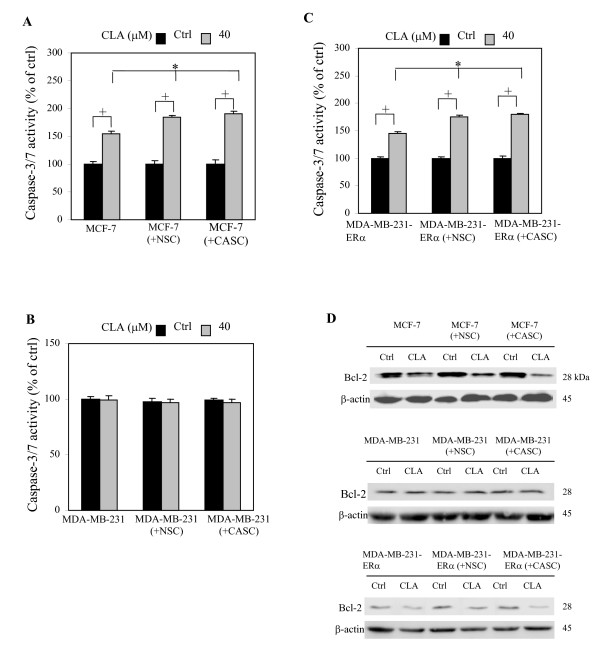
**CLA induced apoptosis of breast cancer epithelial cells is associated with estrogen receptor α (ERα) in co-culture.** MCF-7 (A), MDA-MB-231 (B), ERα transfected MDA-MB-231, and MDA-MB-231-ERα (C), were cultured alone or co-cultured with normal human breast stromal cells (NSC), or co-cultured with cancerous human breast stromal cells (CASC). Cells were treated by *t*10, *c*12-CLA for 7 days and cell apoptosis of MCF-7 (A), MDA-MB-231 (B) or MDA-MB-231-ERα (C) was detected from each group separately by caspase-3/7 activity assay. (D) Bcl-2 protein expression of MCF-7, MDA-MB-231 or MDA-MB-231-ERα was determined by western blot analysis. CLA stands for 40 μM *t*10, *c*12-CLA. Bars represent mean ± SD, n = 3. *p < 0.05 stands for *t*10, *c*12-CLA group comparison of caspase-3/7 activity; +p < 0.05 for control versus *t*10, *c*12-CLA.

We observed that CLA induced apoptosis in ERα(+) breast cells but not in ERα(-) breast cells (Fig. 3). Studies have shown that the local micro-environment is crucial for cancer progression, because cancer epithelial cells are surrounded by various types of stromal cells [28]. The interaction between epithelial cells and extracellular matrix is important for cells to make life or death decisions [29]. To address whether stromal cells can alter CLA-induced apoptosis in ERα(+) and ERα(-) cancer epithelial cells, breast cancer epithelial cell lines, MCF-7, MDA-MB-231, or MDA-MB-231-ERα, were co-cultured with primary human breast stromal cells isolated from normal (NSC) or cancerous (CASC) tissues. After 7 days of CLA treatment, apoptosis in breast cancer epithelial cell lines were measured by caspase-3/7 activity. Apoptosis of co-cultured MCF-7 cells was not changed in comparison with MCF-7 cells cultured alone. CLA treatment induced apoptosis in MCF-7 cells cultured alone and stimulated further apoptosis when these cells were co-cultured with stromal cells (Fig. 6A). A similar pattern of response also was shown in MDA-MB-231-ERα cells (Fig. 6C) but not in MDA-MB-231 cells (Fig. 6B). In agreement with the results in Figs. 6A–C, we observed that Bcl-2 protein was significantly increased in co-cultured MCF-7 and MDA-MB-231-ERα cells but not in MDA-MB-231 cells (Fig. 6D). Furthermore, Bcl-2 expression was decreased in the co-cultured cells treated with CLA. Our results suggest that CLA may modulate the breast microenvironment by blocking the interaction between malignant ERα(+) epithelial cells and stromal cells; although CLA also induce apoptosis of stromal cells (unpublished data), CLA did not induce significant changes in apoptosis in co-cultured MDA-MB-231 cells.

## Discussion

### CLA has anti-estrogenic effects that may correlate with reduced risk of ERα(-) negative breast cancer

Evidence from epidemiological studies has shown that the duration of estrogen exposure is an important risk factor for development of breast cancer. Estrogens are involved in initiation, promotion, and progression of breast carcinogenesis [30]. It has been shown that CLA exerts little to no inhibitory effect on MDA-MB-231 cells compared to MCF-7 cells suggesting the possible involvement of anti-estrogenic effects of CLA on human breast cancers [24,31,32]. Using MCF-7 cells transiently transfected with the estrogen responsive element (ERE), Tanmahasamut et al. (2003) [24] demonstrated that CLA inhibited the promoter's activity directly through the ERE. More recently, Liu and Sidell (2005) [33] showed that CLA suppressed phosphorylation of the estrogen receptor, which may inhibit receptor-ERE interactions in MCF-7 cells. We examined the anti-estrogenic effects of CLA on MCF-7, MDA-MB-231, ERα(+) NEC and ERα(-) NEC (Figs. 1 and 2). CLA had anti-proliferative effects in MCF-7 and ERα(+) NEC but not in MDA-MB-231 and ERα(-) NEC. Potentiation of Tam by combination with CLA was observed in MCF-7 cells. It was observed that CLA reduced cell proliferation in normal mammary gland but did not induce apoptosis of cells within the terminal end buds and lobular epithelium in rats [34-36]. In contrast, CLA induced apoptosis in mammary tumor cells and premalignant rat mammary gland [37]. Our results showed that although CLA also induced apoptosis in ERα(+) NEC, a greater effect was observed in MCF-7 cells than in ERα(+) NEC (Fig. 1D). We speculate that dietary long-term consumption of CLA may have moderate chemopreventive activities in healthy individuals. Furthermore, in breast cancer patients, CLA also might have less of an effect on normal parts of the mammary gland, resulting in a lesser degree of cytotoxicity in these patients.

To ascertain the role of ERα in the anti-estrogenic effects mediated by CLA, MDA-MB-231-ERα cells (ERα transiently transfected MDA-MB-231) were treated with E_2 _or E_2_+CLA. CLA exerted anti-estrogenic effects in MCF-7 and MDA-MB-231-ERα cells but treatment did not induce considerable effects in MDA-MB-231 cells. E_2 _treatment inhibited the growth of MDA-MB-231-ERα and CLA counteracted E_2_-induced growth inhibitory effect (unpublished data). According to these and results shown in Fig. 3, we expect that the anti-cancer activities of CLA will be more effective in ERα(+) breast cancer patients than those with ERα(-) breast cancers. This might explain epidemiological findings showing that CLA intake may be correlated with reduced risk of having an estrogen receptor negative cancer in premenopausal breast cancer patients [20].

### Mechanisms of the anti-estrogenic effects of CLA

The carcinogenic effects of estrogens in breast cancer include the stimulation of breast tissue growth and the protection of cells from apoptosis [30]. A higher proliferative rate in tumors has been correlated with metastasis, death from neoplasia, low disease-free survival rates, and low overall survival rate in human cancer models [31]. Immunohistochemical staining studies suggested that Bcl-2 expression is linked to hormonal regulation in human breast cancers [38]. Interestingly, several studies showed Bcl-2 protein expression in normal as well as hyperplastic and neoplastic breast epithelial cells [38-40]. In agreement with these findings, our results showed that Bcl-2 protein is expressed in both cancerous and normal human breast tissues (Fig. 4). In normal breast tissue samples, 12 out of 14 were from patients under 40 years of age; 10 were diagnosed as macromastia and the remaining four were diagnosed as hypertrophy (Fig. 4). Accordingly, we speculate that Bcl-2 may be important to maintain mammary gland function in these normal patients and Bcl-2 may be involved in the occurrence of mammary hypertrophy and mammary macromastia. It has been suggested that cells express Bcl-2 are immature and may be part of the stem-cell subpopulation in normal mammary gland [40]. Bcl-2 is important for maintenance and development of normal mammary gland. However, Bcl-2 expression is increased when cell proliferation in tissues is dysregulated in hyperplastic and neoplastic disorders. Bcl-2 localization in normal breast tissue may be related to the origin of malignant breast disease [40]. Upon E_2 _stimulation, Bcl-2 protein was increased in ERα(+) normal as well as cancerous human breast (Fig. 5). We have observed that CLA also blocks the estrogenic effects of environmental hormones such as Zeranol, a nonsteroidal agent with estrogenic activity used as a growth promoter in the US beef and veal industries (unpublished data). Taken together, the results suggest that CLA is an excellent candidate for prevention and therapy of estrogen responsive breast cancer.

### CLA blocks the epithelial-stromal interaction in the ERα(+) but not ERα(-) human breast microenvironment

From experimental cancer models, the extracellular microenvironment has been demonstrated to influence tumor formation, the rate of cellular proliferation, the ability of the cancer cells to metastasize and the extent of invasiveness. In many cancers, the influences of the microenvironment are mediated in part by paracrine signaling between epithelial cancer cells and the surrounding stromal cells [41,42]. Although Bcl-2 is expressed highest in malignant epithelial cells, stromal cells also express Bcl-2 but in lesser amounts, which may indicate both autocrine and paracrine effects of Bcl-2 in the tumor microenvironment (Fig. 4). CLA induced more apoptosis in MCF-7 and MDA-MB-231-ERα cells when those cells were co-cultured with stromal cells suggesting that the presence of stromal cells is important in CLA induced apoptosis in ERα(+) cancer cells which support the theory that CLA is incorporated and stored in stromal cells to affect cancer progression [2]. It appeared that CLA was able to interrupt this paracrine signal (Figs. 6A and 6C). However, the involvement of stromal cells did not alter the effects of CLA in MDA-MB-231 cells (Fig. 6B). Although CLA possesses apoptotic effect in both normal and cancerous stromal cells (data not shown), cancerous ERα negative breast epithelial cells may acquire independent self-regulation from the surrounding stromal compartment.

### Efficacy of CLA in breast cancer chemoprevention

Preclinical studies [43,44] showed promising anti-mammary tumor activity in mouse (~3.5 mg/20 g mouse · d) and rat models (~13 mg/275 g rat · d) that are equivalent to the effective concentration in humans (~2.8 g CLA/70 kg · d). Epidemiological studies have demonstrated that a diet composed of CLA-rich foods (204 mg CLA/day), particularly cheese, may protect against breast cancer in postmenopausal women [45]. More recently, another study showed that CLA intake (134–155 mg CLA/day) was not related to overall breast cancer risk, however, the risk of estrogen receptor (ER)-negative breast cancer among premenopausal women was reduced [20]. It is clear that women consume ~100 times less than the amount of CLA estimated to provide protection. A moderate but effective influence may occur after low level long term consumption of CLA. Since CLA is a natural by-product of rumen fermentation and is found in foods derived from ruminants, consumption of CLA from foods for the purposes of chemoprevention likely will not affect the safety of consumers. It is interested to note that special diet of cow is able to increase CLA in milk fat [2]. Whether CLA will cause any toxic effects on these cows? We speculate that CLA will be less likely to induce cell apoptosis in the udder of these cows because CLA in the circulation will not reach the doses used in *in vitro *cell culture models nor the concentrations of CLA-enriched diet in animal studies.

## Conclusion

We investigated the role of ERα in CLA induced apoptosis in human breast tissue. Treatment of ERα(+) breast cancer cells and tissue with E_2 _led to stimulated growth and up-regulated Bcl-2 expression; however, these carcinogenic effects were diminished by either CLA or Tam and were suppressed further by the combination of CLA and Tam. By contrast, ERα(-) cells and tissue failed to respond to the treatments. This result was further confirmed by the transfection of ERα into these cells which restored their response to CLA. Therefore, our findings demonstrate that CLA exert anti-estrogenic effects and ERα plays important roles in CLA induced apoptosis in human breast tissues. Future study examine the knock down of ERα in MCF-7 cells to confirm the loss of response of these cells to CLA are crucial to support the proposed mechanism – ERα plays important role in CLA's anti-cancer activity.

## Competing interests

The authors declare that they have no competing interests.

## Authors' contributions

L–SW carried out most of the experiment and drafted the manuscript. Y–WH carried out the western blot analysis. SL and PY participated in revising the manuscript. L–SW is under the direct advising of YCL. All authors read and approved the final manuscript.

## List of abbreviations used

CLA: conjugated linoleic acid; E_2_: 17β-estradiol; ERα(+): estrogen receptor α positive; ERα(-): estrogen receptor α negative; ERE: estrogen responsive element; VEGF: vascular endothelial growth factor; PTPγ: protein tyrosine phosphatase γ; ATAC: Arimidex, Tamoxifen, Alone or in Combination; ASCO: American Society of Clinical Oncology; DCC: Dextran-Coated Charcoal; FBS: fetal bovine serum; NEC: primary cultured non-cancerous human breast epithelial cells; NSC: primary cultured non-cancerous human breast stromal cells; CAEC: primary cultured cancerous human breast epithelial cells; CASC: primary cultured cancerous human breast stromal cells; Tam: 4-Hydroxytamoxifen.

## Pre-publication history

The pre-publication history for this paper can be accessed here:


